# Involvement of cortico-efferent tracts in flail arm syndrome: a tract-of-interest-based DTI study

**DOI:** 10.1007/s00415-021-10854-6

**Published:** 2021-10-21

**Authors:** Angela Rosenbohm, Kelly Del Tredici, Heiko Braak, Hans-Jürgen Huppertz, Albert C. Ludolph, Hans-Peter Müller, Jan Kassubek

**Affiliations:** 1 Department of Neurology, University of Ulm, Oberer Eselsberg 45, 89081 Ulm, Germany; 2 Swiss Epilepsy Clinic, Klinik Lengg, Zurich, Switzerland; 3 Deutsches Zentrum für Neurodegenerative Erkrankungen (DZNE), Ulm, Germany

**Keywords:** Amyotrophic lateral sclerosis, Diffusion tensor imaging, Flail arm syndrome, Magnetic resonance imaging, Motor neuron disease

## Abstract

**Background:**

Flail arm syndrome is a restricted phenotype of motor neuron disease that is characterized by progressive, predominantly proximal weakness and atrophy of the upper limbs.

**Objective:**

The study was designed to investigate specific white matter alterations in diffusion tensor imaging (DTI) data from flail arm syndrome patients using a hypothesis-guided tract-of-interest-based approach to identify in vivo microstructural changes according to a neuropathologically defined amyotrophic lateral sclerosis (ALS)-related pathology of the cortico-efferent tracts.

**Methods:**

DTI-based white matter mapping was performed both by an unbiased voxel-wise statistical comparison and by a hypothesis-guided tract-wise analysis of fractional anisotropy (FA) maps according to the neuropathological ALS-propagation pattern for 43 flail arm syndrome patients vs 43 ‘classical’ ALS patients vs 40 matched controls.

**Results:**

The analysis of white matter integrity demonstrated regional FA reductions for the flail arm syndrome group predominantly along the CST. In the tract-specific analysis according to the proposed sequential cerebral pathology pattern of ALS, the flail arm syndrome patients showed significant alterations of the specific tract systems that were identical to ‘classical’ ALS if compared to controls*.*

**Conclusions:**

The DTI study including the tract-of-interest-based analysis showed a microstructural involvement pattern in the brains of flail arm syndrome patients, supporting the hypothesis that flail arm syndrome is a phenotypical variant of ALS.

**Supplementary Information:**

The online version contains supplementary material available at 10.1007/s00415-021-10854-6.

## Introduction

The neuropathological process underlying amyotrophic lateral sclerosis (ALS) entails abnormal changes of the endogenous and predominantly intranuclear protein TDP-43 (transactive response DNA-binding protein 43) which progress at different rates, but in a similar sequence of four stages, in ALS patients’ brains [1, 2]. Clinically, ALS patients may present with phenotypes that differ from the ‘classical’ involvement of the upper and the lower motor neuron (UMN, LMN) according to established diagnostic criteria: on the one hand, the diagnostic criteria for primary lateral sclerosis as a slowly progressive upper motor neuron syndrome have been recently updated [3], whereas, on the other hand, it is recognized that upper motor neuron signs are not always clinically evident [4], such as in progressive muscular atrophy or progressive bulbar palsy. This concept of ‘restricted’ phenotypes also includes flail arm syndrome, which often begins with asymmetric predominantly proximal deficits of the arms [5]. The term flail arm syndrome was introduced in the literature in 1998 [6] for a subgroup of ALS patients with signs of lower motor neuron disease confined to the shoulder and the proximal or distal portion of the arms and little or no involvement of other muscles in early disease stages, probably identical to the Vulpian Bernhardt syndrome, as described more than a century earlier [7]. Operational definitions with standardized criteria for flail arm syndrome have been proposed [8], which later were confirmed by further clinical and epidemiological data [9, 10].

Recently, structural and functional neuroimaging findings have greatly modified longstanding notions regarding the pathophysiology of ALS [11, 12]. For the in vivo analysis of the proposed sequential cerebral pathology pattern according to the neuropathological staging concept of ALS, a dedicated magnetic resonance imaging (MRI) approach exists that uses a tract-of-interest (TOI)-based analysis technique of diffusion tensor imaging (DTI) to demonstrate ALS-specific four-stage cortico-efferent tract pathology [13, 14]. Since the neuropathological confirmation of cerebral TDP43 pathology within the complete spectrum of ALS phenotypes is limited by the lack of autopsies, the MRI-based staging technique has been applied to patients with progressive lower motor neuron disease [15, 16], with PLS [17, 18], and with progressive bulbar palsy [19]. The same tract involvement patterns as those seen in ´classical´ ALS have been demonstrated in all of these restricted phenotypes [20]. In the current study, TOI-based DTI analysis was performed in a group of patients with the clinical presentation of flail arm syndrome to support the hypothesis that these patients, like the other restricted phenotypes, show the same microstructural involvement patterns as in ALS.

## Methods

### Subjects and patient characteristics

Forty-three flail arm syndrome patients (32 males, 11 females) were included who met the diagnostic criteria for flail arm syndrome as proposed by Wijesekera and colleagues [8]. Flail arm syndrome was diagnosed in patients with paresis of both upper limbs and without bulbar and lower limbs symptoms during a time period of 12 months after their visit, as previously described [9]. To be eligible, subjects had to fulfill the following criteria: no clinical diagnosis of frontotemporal dementia (FTD), no mutations of major genes related to motor neuron disease (if known), and no other major systemic, psychiatric or neurological illnesses. Further mandatory criteria for inclusion were negative tests for other neuromuscular diseases and for infections of the central nervous system, and the presence of routine MRI scans that excluded any brain or spine abnormalities which might indicate a different etiology of the clinical symptoms. Mean disease duration in the flail arm syndrome group was 20 months (range 4 to 66 months), and age of onset of the motor disorder was 62 ± 11 years. All patients underwent standardized clinical, neurological, and routine laboratory examinations; 40/43 were under medication with riluzole. In this group, 40 patients presented with bilateral paresis in the arms at date of MRI, while unilateral arm symptoms at date of MRI were observed in 3 patients. Further follow-up after MRI was lost in 13/43 patients, 4 progressed to the bulbar region and 19 to the lumbar region, while 7 patients showed no spreading to other body regions at the time of data analysis. At the time of data analysis, 12 patients had died with a mean survival of 40.6 ± 12.4 months. Flail arm syndrome patients presented with a revised ALS functional rating scale (ALS-FRS-R) [21] of 41 ± 6 on average. Median slope of ALS-FRS-R reduction over disease duration was − 0.43/month (90th percentile, range − 0.08; − 2.03).

The flail arm syndrome patients were compared with a group of 43 ‘classical’ ALS patients (29 male/14 female, age 64 ± 11 years) and with a group of 40 age- and gender-matched controls. Gross brain pathology, including vascular brain alterations, was excluded by conventional MRI. All control individuals lacked a family history of neuromuscular disease and had no history of neurologic, psychiatric, or other major medical illnesses, and were recruited among spouses of patients and by word-of-mouth.

In the ‘classical’ ALS group, onset was lumbar in 50%, cervical in 30%, and bulbar in 20%. At the time of data analysis, 24 patients had died with a mean survival of 30.2 ± 15.8 months. In the subgroup analysis, survival was 24.3 ± 6.6 months in upper limb onset (not fulfilling the criteria of flail arm syndrome) and 35.7 ± 19.5 months in lower limb onset. Median slope of ALS-FRS-R reduction over disease duration was − 0.67/month (90th percentile range − 0.19; − 1.90). Since survival could be calculated from a deceased subgroup only (flail arm syndrome, *n* = 13, ‘classical’ ALS, *n* = 24), we decided to statistically compare the clinical progression of these two cohorts by ALS-FRS-R slope per months (see Table 1). The progression rate in the ALS group was higher compared to the flail arm syndrome group, however, not significant.

**Table 1:** Subjects’ characteristics

	Flail arm syndrome (*n* = 43)	‘Classical’ ALS (*n* = 43)	Controls (*n* = 40)	*p*
Male/female	32/11	29/14	25/15	Kruskal–Wallis: 0.6
Age/years (mean ± std. dev.)	64 ± 11	64 ± 11	61 ± 14	Kruskal–Wallis: 0.7
ALS-FRS-R	41 ± 6	40 ± 6	–	*t*-test: 0.4
Disease duration/months (mean ± std. dev.)	20 ± 14	19 ± 15	–	*t*-test: 0.7
ALS-FRS-R monthly slope median (range 90th percentile)	− 0.43 (− 0.08; − 2.03)	− 0.67 (− 0.19; − 1.90)	–	*t*-test: 0.1

*ALS-FRS-R* revised ALS functional rating scale

All participants provided written informed consent for the study protocol according to institutional guidelines which had been approved by the Ethics Committee of Ulm University, Germany (No. 19/12).

A summary of the participants’ characteristics is given in Table 1. The group comparison concerning age and gender by *t*-test showed no significant differences.

### MRI acquisition

MRI scanning was performed on a 1.5 Tesla Magnetom Symphony (Siemens Medical, Erlangen, Germany); the DTI study protocol [echo time (TE)/repetition time (TR), 95 ms/8000 ms] contained 52 volumes (128 × 128 × 64 voxels, voxel size 2.0 mm × 2.0 mm × 2.8 mm), including 48 gradient directions with *b* = 1000 s/mm^2^ and four gradient directions with *b* = 0. The study protocol consisted of a T1-weighted 3-dimensional scan with 144 slices, TE/TR 4.2 ms/1640 ms, 256 × 256 pixels, slice thickness 1.2 mm, pixel size 1.0 mm × 1.0 mm.

### Data analysis

#### DTI data analysis

The software *Tensor Imaging and Fiber Tracking* (TIFT) [22] was used for data analysis; details of the algorithms have been described in detail previously [13, 14, 23]. After stereotaxic normalization to the Montreal Neurological Institute (MNI) space, fractional anisotropy (FA), axial diffusivity (AD), and radial diffusivity (RD) maps were calculated as DTI metrics to analyze white matter microstructure [24]. While FA is sensitive to microstructural changes, it does not indicate a specific type of lesion; on the other hand, AD tends to be strongly affected by axonal injury whereas RD is sensitive to white matter damage due to demyelination and less to changes in the axonal density or size [25–27]. Prior to correction for the covariate age, a Gaussian filter of 8 mm full width at half maximum was applied for smoothing of FA, AD, and RD maps.

Voxel-wise statistical comparison of the DTI metrics was performed by Student’s t-test to detect alterations between the subject groups (whole brain-based spatial statistics, WBSS); voxels with FA values below 0.2 were not considered for statistical comparison, since cortical grey matter shows FA values up to 0.2 [28]. Correction for multiple comparisons was performed using the false-discovery-rate (FDR) algorithm at *p* < 0.05 [29]; further reduction of the alpha error was performed by a spatial correlation algorithm eliminating small isolated groups of voxels in the size range of the smoothing kernel (8 mm) leading to a threshold cluster size of 256 voxels.

The tract-of interest (TOI) approach [13, 14, 16–18] defined tracts for the four ALS stages [1, 30], i.e. the corticospinal tract (CST, representative of ALS stage 1), the corticorubral and corticopontine tracts (corresponding to ALS stage 2), the corticostriatal pathway (corresponding to ALS stage 3), and the proximal perforant path (corresponding to ALS stage 4). A tract originating from the corpus callosum (CC) area V was used as reference where no involvement in ALS-associated neurodegeneration could be anticipated. To identify alterations along these tracts, tract-wise fractional anisotropy statistics (TFAS) [31] was performed by statistically comparing the FA values in the respective tracts between two subject groups by Student’s *t*-test for FA values ≥ 0.2 (see above); as the subject groups were large enough to show a Gaussian distribution of FA values, the use of Student’s *t*-test was justified.

#### Atlas-based volumetry

The grey matter volume in the precentral gyrus was assessed by atlas-based volumetry (ABV), an automated and observer-independent voxel-based image processing method using algorithms of the SPM12 software (Wellcome Trust Centre for Neuroimaging, London, UK, www.fil.ion.ucl.ac.uk/spm) and predefined masks from the LONI Probabilistic Brain Atlas to determine volumes of various brain structures at individual subject level from T1-weighted 3-dimensional structural images [32, 33].

## Results

### Whole brain-based spatial statistics

**Fig. 1:**
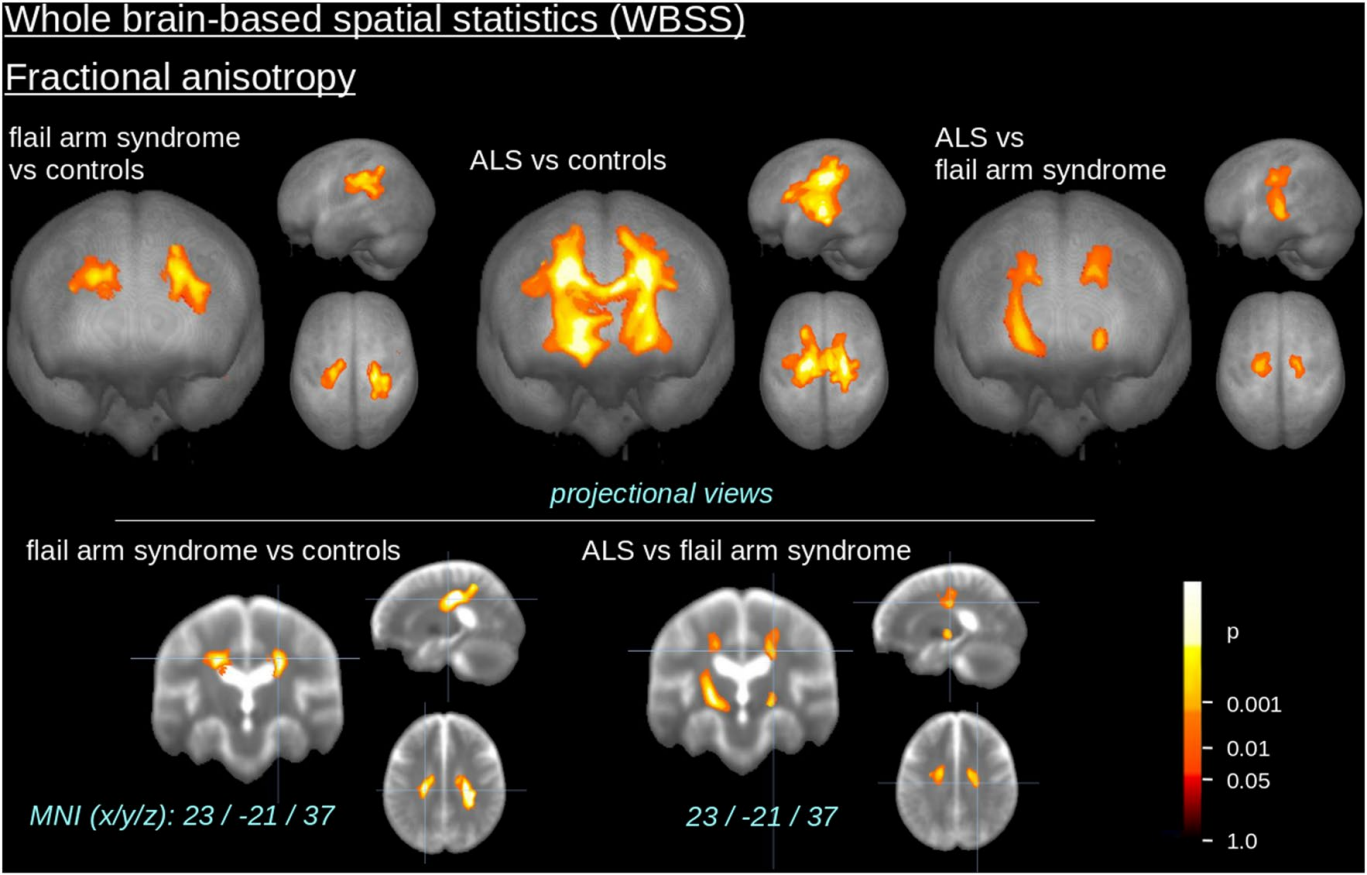
Whole brain-based spatial statistics (WBSS) of FA maps of flail arm syndrome patients and ALS patients vs controls. WBSS of FA maps [*p* < 0.05, false-discovery-rate (FDR) corrected] demonstrated clusters of regional FA reductions for flail arm syndrome patients vs controls as well as for ALS patients vs controls predominantly along the corticospinal tract (CST)

The comparison at the group level by WBSS of FA maps for flail arm syndrome patients *vs* controls demonstrated several clusters of regional alterations at *p* < 0.05 (corrected for multiple comparisons). Maps of FA reductions are depicted in Fig. 1 for the group comparisons. When comparing flail arm syndrome patients and controls, regional FA reduction was observe in the upper CST (corresponding to neuropathological stage 1 of ALS); the identical FA reduction pattern was observed in the comparison of ALS patients vs controls. The comparison of flail arm syndrome patients and ALS patients revealed only small quantitative differences bihemispherically along the central and upper CST. A summary of all significant clusters at the group level is provided in Table 2.

**Table 2:** Cluster results of WBSS of FA maps (thresholded at FDR-corrected *p* < 0.05)

Fractional anisotropy (FA)					
No.	Size/mm^3^	MNI of maximum (*x y z*)	Hemisphere	Average *p *(FDR-corrected)	Anatomical localization (maximum)
*Flail arm syndrome vs. controls*					
1	9426	35 − 34 28	R	< 0.000001	Upper CST
2	5338	− 23 − 24 35	L	< 0.000001	Upper CST
*ALS vs. controls*					
3	74,253	− 24 − 17 5	R/L	< 0.000001	CST
*Flail arm syndrome vs. ALS*					
4	8561	− 24 − 19 4	L	0.000002	Central CST/upper CST
5	3541	16 − 12 39	R	0.000004	Upper CST
6	707	21 − 17 0	R	0.000001	Central CST

*MNI* Montreal Neurological Institute brain atlas, *FDR* false-discovery rate, *CST* corticospinal tract

**Fig. 2:**
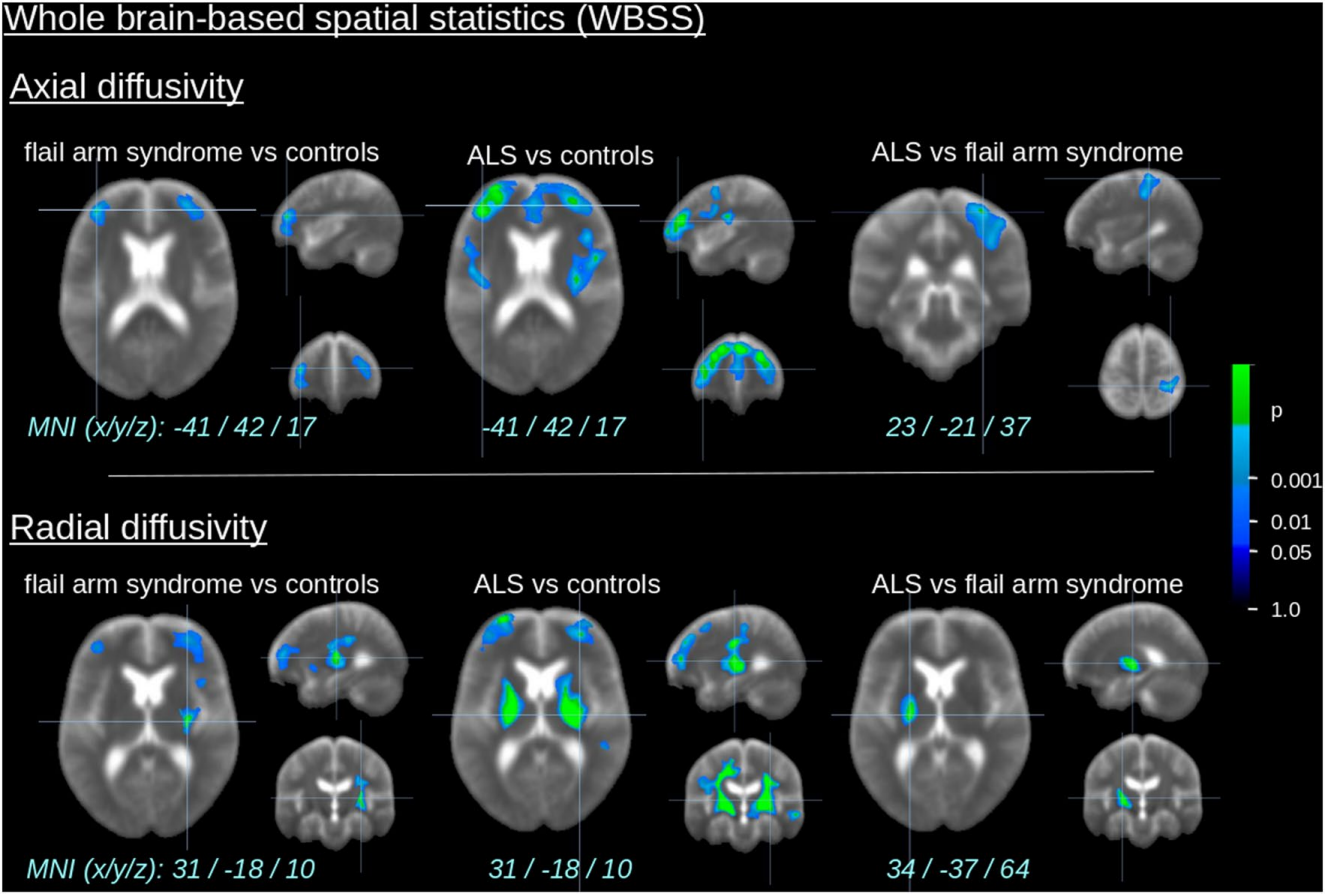
Whole brain-based spatial statistics (WBSS) of AD and RD maps of flail arm syndrome patients and ALS patients vs controls. WBSS of AD and RD maps [*p* < 0.05, false-discovery-rate (FDR) corrected] demonstrated clusters of regional increase for flail arm syndrome patients *vs* controls as well as for ALS patients *vs* controls predominantly the frontal lobes and along the corticospinal tract (CST)

The comparison at the group level by WBSS of AD and RD maps for the flail arm syndrome patients *vs* controls demonstrated several clusters of regional alterations at *p* < 0.05 (corrected for multiple comparisons) (Fig. 2), especially regional AD and RD increase bihemispherically in the frontal lobes and RD increase in the CST. A regionally similar but more pronounced AD and RD increase pattern was observed in the comparison of ‘classical’ ALS patients *vs* controls. The comparison between flail arm syndrome patients and ALS patients revealed only small quantitative differences along the central and upper CST. A summary of all significant clusters at the group level is provided in Supplementary Table 1.

### Differences in the specific tract systems

**Fig. 3:**
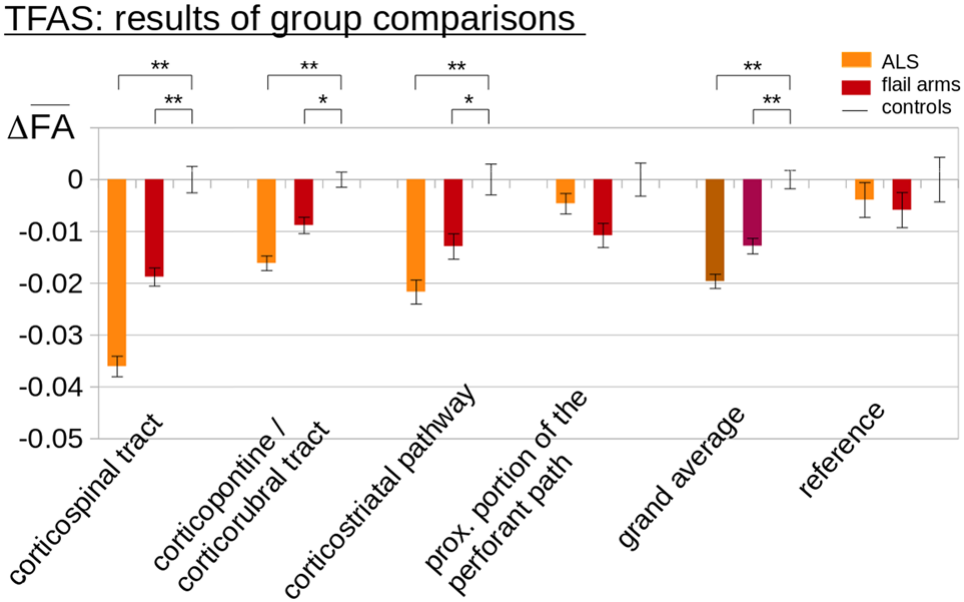
Tractwise fractional anisotropy statistics (TFAS) of FA maps at the group level for flail arm syndrome patients and controls. TFAS demonstrated significant regional FA reductions in ALS-related tract systems and in the grand average between flail arm syndrome patients and controls as well as between ALS patients and controls. No significant alterations between groups were observed in the reference tract. Error bars are the standard error of the mean (SEM). *p < 0.05, **p < 0.001

The hypothesis-guided analysis of the FA differences in the ALS-related tracts using TFAS showed significant differences of the averaged FA values between the flail arm syndrome group and the control group, with the most prominent FA alterations in the CST (i.e., the tract related to ALS stage 1), followed by FA reductions in the corticorubral and corticopontine tracts, and in the corticostriatal pathway (i.e., the tracts related to ALS stages 2 and 3) (Fig. 3), a FA reduction (which was not significant) was observed in the proximal portion of the perforant path (i.e., the tract related to ALS stage 4). For the grand average of the stage-related tract systems, significant FA reductions were observed for flail arm syndrome patients compared to controls. No significant FA alterations were found if group comparisons for the reference paths were performed. The identical tract-based related pattern could be shown when comparing the ‘classical’ ALS patients *vs* controls.

### ALS-related staging at the individual level

**Fig. 4:**
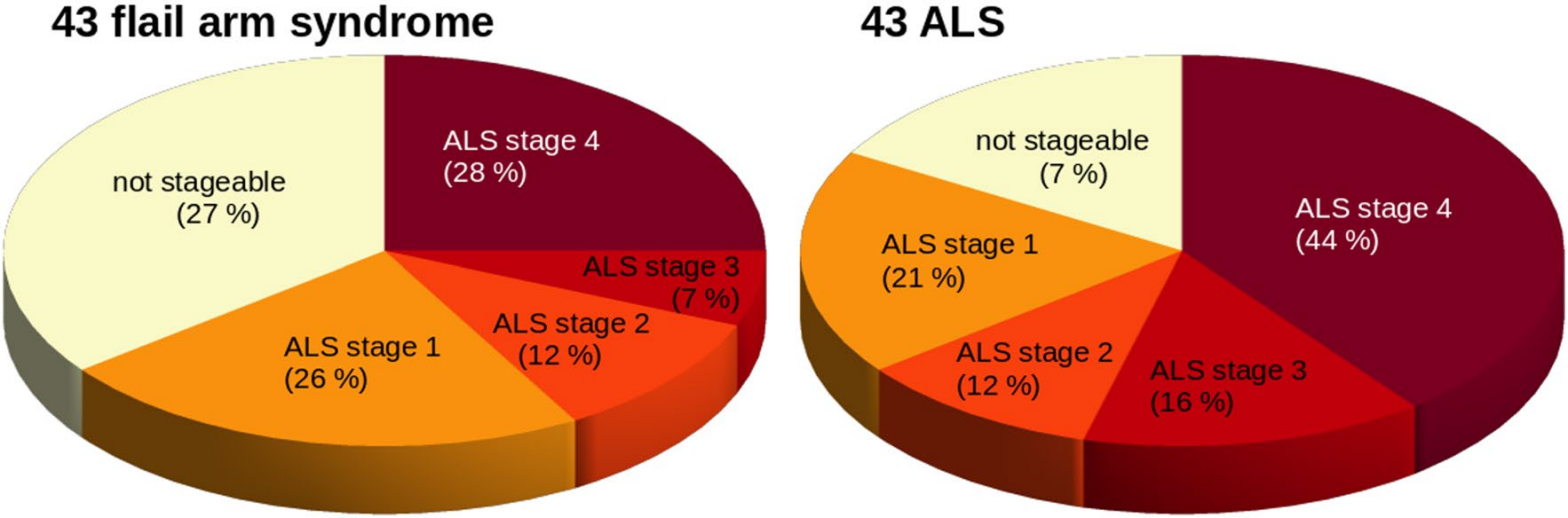
Distribution of staging categorization in flail arm syndrome patients and ‘classical’ ALS patients

When the (FA based) ALS staging categorization was performed for the flail arm syndrome patients, 69% could be categorized into ALS stages, similar to previous studies [14]. The distribution of ALS stages for flail arm syndrome patients was as follows: 26% were in ALS stage 1, 12% in ALS stage 2, 7% in ALS stage 3, and 28% in ALS stage 4 (Fig. 4). A similar distribution could be observed for the ALS patients of whom 90% could be categorized into ALS stages: 21% were in ALS stage 1, 12% in ALS stage 2, 16% in ALS stage 3, and 44% in ALS stage 4.

### Atlas-based volumetry

**Fig. 5:**
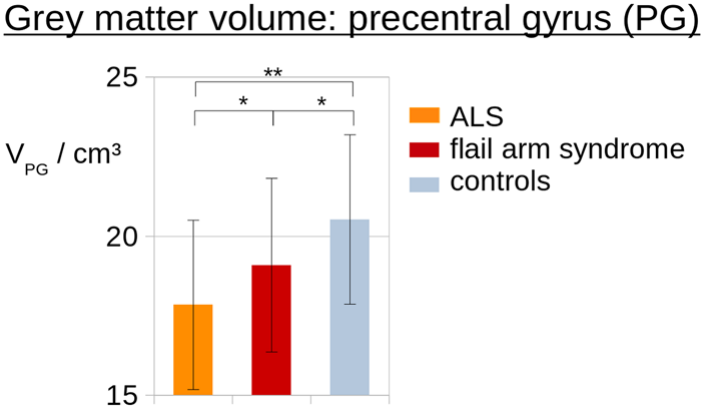
Atlas-based volumetry of the precentral gyrus. The precentral gyrus shows grey matter atrophy both in ALS and in flail arm syndrome patients compared to controls. Atrophy was more pronounced in the ‘classical’ ALS patients. *p < 0.05, **p < 0.005

Region-wise comparisons of ABV data were performed by normalizing all individual volume results for each investigated brain area to the intracranial volume (ICV). The volume of the precentral gyrus was analysed as a measure of the cortical atrophy pattern of the motor system. Both in ‘classical’ ALS and in flail arm syndrome patients, the volume of the precentral gyrus was atrophied compared to controls, with more pronounced atrophy in ALS patients (Fig. 5).

## Discussion

The flail arm syndrome (also known as the Vulpian Bernhardt variant and sometimes referred to as man-in-the barrel syndrome or brachial amyotrophic diplegia [9]) is clinically defined as an initially localized variant with a mainly LMN presentation; it is dominated by severe atrophy of the proximal or distal upper limbs with the arms hanging flaccidly on either side [34]. The clinical management of this motor neuron disease is compromised by a delay in diagnosis and a high rate of initial misdiagnoses [9]. Technical markers, such as neuroimaging, might serve as guides in the diagnostic classification beyond clinical recognition and help to identify flail arm syndrome as a sub-form of ALS.

Our DTI study in a large group of flail arm syndrome patients displayed central nervous system involvement of the motor system, in particular clinically covert involvement of the pyramidal tract (CST), identical to that in the patients with ‘classical’ ALS. In detail, the unbiased WBSS analysis of all significant changes in the brain at the group level demonstrated a pattern in flail arm syndrome that included mainly the CST as the anatomical structure corresponding to ALS stage 1 in agreement with previous data in ALS, including a meta-analysis of DTI studies [35]. More specifically, in vivo neuropathological staging by TOI-based DTI analysis [13, 14] showed that patients with flail arm syndrome had a pattern of microstructural alterations in corticofugal tracts identical to those seen in ALS. The observation that patients with the flail arm syndrome phenotype showed the same tract involvement as ‘classical’ ALS according to the proposed neuropathologically defined cerebral propagation scheme supports the hypothesis that flail arm syndrome shares the cerebral involvement pattern with ALS and is, as such, an ALS variant. The demonstration of the involvement of the cerebral tracts according to the neuropathological ALS-propagation pattern supports previous studies which showed corticospinal tract pathology in pure lower motor neuron variants of ALS [36, 37].

Thus, on the basis of this neuroimaging study, we believe that the proposed staging scheme for ALS [1] is also valid for flail arm syndrome patients who should, as a consequence, receive appropriate therapies, including the same access to health care systems as ALS patients and the opportunity to be enrolled into one of the growing number of clinical trials for this indication. This conclusion is supported by the fact that the patient group in the present imaging study is representative of the flail arm syndrome phenotype, including male preponderance and a slower disease course (Table 1). Our findings are an extension of the application of the DTI-based in vivo neuropathological staging to restricted ALS phenotypes with the demonstration of corticofugal tract involvement according to the established TDP-43 propagation scheme, as previously performed in progressive lower motor neuron disease [15, 16], PLS [17, 18], and progressive bulbar palsy [19]. All of these TOI-based FA mapping-based studies strongly support the classification of all the restricted phenotypes [5] as ALS variants, with the identical cerebral damage pattern as defined for ALS [20]. In the neuroimaging domain, the inclusion of flail arm syndrome patients into multisite studies with harmonized protocols [23, 38] for the development of imaging-based biological markers is also supported by the identification of the same brain involvement pattern as in ‘classical’ ALS.

Disease deterioration was more pronounced in the ‘classical ALS’ comparison group with a median survival of 30 months—flail arm syndrome patients showed a prolonged survival around 40 months (deceased subgroup, *N* = 13) and a trend for lower ALS-FRS-R slope. Most of the flail arm syndrome patients further progressed to other body regions (mainly to the lower limbs).

This study was not without limitations. First, the study used cross-sectional data so that longitudinal investigations in flail arm syndrome have to await future studies. Second, neuropathological confirmation of the TDP-43 propagation scheme in the central nervous system by autopsy results was not available.

In summary, the tract-of-interest-based DTI analysis demonstrated in vivo the same stereotypical pathoanatomical patterns in the brains of flail arm syndrome and ´classical´ ALS patients, thereby confirming the clinical approach that this syndrome is a restricted phenotypical variant of ALS [10], in accordance with the latest proposal for a revision of the El Escorial criteria [5, 39] and with a current proposal for clinical diagnosis [4]. Ultimately, these findings should encourage future studies across the phenotypical spectrum of ALS to contribute to our understanding of potential modifiers of the clinical presentations in ALS.

## Supplementary Information

Below is the link to the electronic supplementary material.

Supplementary file1 (DOCX 17 KB)

## Data Availability

The original contributions presented in the study are included in the article, further inquiries can be directed to the corresponding author/s.
