# Supplementary material for: Involvement of cortico-efferent tracts in flail arm syndrome: a tract-of-interest-based DTI study
Source: J Neurol. 2021 Oct 21;269(5):2619–26. doi: 10.1007/s00415-021-10854-6 (PMC9021061; doi:10.1007/s00415-021-10854-6)
Supplement: Supplementary file 1 — Supplementary file1 (DOCX 17 KB) [file 415_2021_10854_MOESM1_ESM.docx]

| **no.** | **size / mm^3^** | **MNI of maximum**  **(*x y z*)** | **hemisphere** | **average p**  **(FDR-corrected)** | **anatomical localization**  **(maximum)** |
| --- | --- | --- | --- | --- | --- |
| **Axial diffusivity (AD)** | | | | | |
| **flail arm syndrome vs. controls** | | | | | |
| 7 | 4,772 | 31 46 15 | R | < 0.000001 | frontal lobe |
| 8 | 4,251 | -41 42 15 | L | < 0.000001 | frontal lobe |
| **`classical` ALS vs. controls** | | | | | |
| 9 | 36,431 | -31 58 3 | L | < 0.000001 | frontal lobe |
| 10 | 35,435 | 31 58 3 | R | < 0.000001 | frontal lobe |
| 11 | 25,569 | 48 -2 19 | R | < 0.000001 | central CST |
| 12 | 17,756 | -48 -9 20 | L | < 0.000001 | central CST |
| **flail arm syndrome vs. `classical` ALS** | | | | | |
| 13 | 9,578 | 34 -37 64 | R | 0.000002 | upper CST |
| **Radial diffusivity (RD)** | | | | | |
| **flail arm syndrome vs. controls** | | | | | |
| 14 | 9,536 | 31 -17 11 | R | < 0.000001 | CST |
| 15 | 7,686 | 31 46 14 | R | < 0.000001 | frontal lobe |
| 16 | 3,875 | -38 37 14 | L | < 0.000001 | frontal lobe |
| **`classical` ALS vs. controls** | | | | | |
| 17 | 36,941 | -30 59 5 | L | < 0.000001 | frontal lobe |
| 18 | 35,341 | -25 -20 8 | L | < 0.000001 | CST |
| 19 | 30,161 | 24 -20 9 | R | < 0.000007 | CST |
| 20 | 8,835 | 28 42 33 | R | < 0.000007 | frontal lobe |
| **flail arm syndrome vs. `classical` ALS** | | | | | |
| 21 | 8,211 | 30 -38 65 | R | < 0.000001 | upper CST |
| 22 | 4,332 | -21 -19 1 | L | < 0.000001 | central CST |

**Supplementary Table 1: Cluster results of WBSS of AD and RD maps (thresholded at FDR-corrected p < 0.05).** MNI, Montreal Neurological Institute brain atlas; FDR, false discovery rate; CST, corticospinal tract.
